# Oxygen-Based Autoregulation Indices Associated with Clinical Outcomes and Spreading Depolarization in Aneurysmal Subarachnoid Hemorrhage

**DOI:** 10.1007/s12028-024-02088-x

**Published:** 2024-08-27

**Authors:** Andrew P. Carlson, Thomas Jones, Yiliang Zhu, Masoom Desai, Ali Alsarah, C. William Shuttleworth

**Affiliations:** 1 Department of Neurosurgery, University of Virginia School of Medicine, Charlottesville, VA USA; 2 Department of Neurosurgery, University of New Mexico School of Medicine, Albuquerque, NM USA; 3 Department of Psychiatry, University of New Mexico School of Medicine, Albuquerque, NM USA; 4 Department of Internal Medicine, University of New Mexico School of Medicine, Albuquerque, NM USA; 5 Department of Neurology, University of New Mexico School of Medicine, Albuquerque, NM USA; 6 Department of Neurology, Harvard University, Boston, MA USA; 7 Department of Neuroscience, University of New Mexico School of Medicine, Albuquerque, NM USA

**Keywords:** Subarachnoid hemorrhage, Delayed cerebral ischemia, Spreading depolarization, Cerebral autoregulation, Cerebral ischemia

## Abstract

**Background:**

Impairment in cerebral autoregulation has been proposed as a potentially targetable factor in patients with aneurysmal subarachnoid hemorrhage (aSAH); however, there are different continuous measures that can be used to calculate the state of autoregulation. In addition, it has previously been proposed that there may be an association of impaired autoregulation with the occurrence of spreading depolarization (SD) events.

**Methods:**

Study participants with invasive multimodal monitoring and aSAH were enrolled in an observational study. Autoregulation indices were prospectively calculated from this database as a 10 s moving correlation coefficient between various cerebral blood flow (CBF) surrogates and mean arterial pressure (MAP). In study participants with subdural electrocorticography (ECoG) monitoring, SD was also scored. Associations between clinical outcomes using the modified Rankin scale and occurrence of either isolated or clustered SD were assessed.

**Results:**

A total of 320 study participants were included, 47 of whom also had ECoG SD monitoring. As expected, baseline severity factors, such as modified Fisher scale score and World Federation of Neurosurgical Societies scale grade, were strongly associated with the clinical outcome. SD probability was related to blood pressure in a triphasic pattern, with a linear increase in probability below MAP of ~ 100 mm Hg. Multiple autoregulation indices were available for review based on moving correlations between mean arterial pressure (MAP) and various surrogates of cerebral blood flow (CBF). We calculated the pressure reactivity (PRx) using two different sources for intracranial pressure (ICP). We calculated the oxygen reactivity (ORx) using the partial pressure of brain tissue oxygen (PbtO_2_) from the Licox probe. We calculated the cerebral blood flow reactivity (CBFRx) using perfusion measurements from the Bowman perfusion probe. Finally, we calculated the cerebral oxygen saturation reactivity (OSRx) using regional cerebral oxygen saturation measured by near-infrared spectroscopy from the INVOS sensors. Only worse ORx and OSRx were associated with worse clinical outcomes. Both ORx and OSRx also were found to increase in the hour prior to SD for both sporadic and clustered SD.

**Conclusions:**

Impairment in autoregulation in aSAH is associated with worse clinical outcomes and occurrence of SD when using ORx and OSRx. Impaired autoregulation precedes SD occurrence. Targeting the optimal MAP or cerebral perfusion pressure in patients with aSAH should use ORx and/or OSRx as the input function rather than intracranial pressure.

## Introduction

Management of delayed cerebral ischemia (DCI) [1] after aneurysmal subarachnoid hemorrhage (aSAH) remains both a significant challenge and one of the major targetable secondary injury mechanisms in the neurointensive care unit. Evolving understanding of the mechanisms of DCI has opened new physiologically based therapeutic approaches to prevent and treat this problem. Two of the most relevant of these include the role of impaired autoregulation [2–9] and occurrence of spreading depolarization (SD) events [7, 10–13].

Cerebral autoregulation (CA) is the adaptive mechanism by which the brain maintains constant cerebral blood flow (CBF) over a wide range of mean arterial pressure (MAP) [14]. In cases of injury, autoregulation can be impaired, shifted, or even lost such that moderately low blood pressure could lead to potentially ischemic levels of blood flow and moderate elevations could lead to hyperemia, elevated intracranial pressure (ICP), and secondary damage [3, 15]. There is no perfect tool to measure autoregulation, especially in a condition such as aSAH, in which there can be significant temporal and regional changes in autoregulation the weeks following the initial bleed [16]. Continuous indices that use a rolling correlation coefficient between MAP and various surrogates for CBF have been developed to gain insight into the autoregulatory status and how it evolves over the course of admission [16–18]. A target that minimizes the index can then be hypothesized on a patient-specific basis and potentially be used to refine the patient-specific optimum MAP (MAPopt) or optimum cerebral perfusion pressure (CPPopt) [19].

SDs are massive, nonsynaptically mediated, slow-moving depolarizing events that are electrophysiologically very similar to terminal depolarization/brain death, so they can be considered a type of “near-death event” [20–22], especially when occurring in metabolically compromised regions. When there is adequate metabolic substrate (blood flow, glucose, oxygen), tissue can slowly recover over minutes to hours, beginning neuronal transmission after a transient period of minutes [23]. However, in extremely metabolically compromised tissue, SD can result in expansion of ischemia due to the large metabolic requirements for recovery [22, 23]. This process is likely cyclical, in which in vulnerable tissue, metabolic transients, such as hypoxia, hypotension, and even cortical excitation, can trigger SD [24–26] and SD can, in turn, further stress this metabolically compromised tissue, resulting in expansion of ischemia [27]. In aSAH, the occurrence of SD has been strongly associated with worse clinical outcomes and episodes of neurologic deficit [13].

An important factor contributing to initiation of SD is inadequate CBF [28, 29]. In conditions of impaired autoregulation, in which CBF may fall into ischemic zones and trigger SD, even in the normal MAP range, it would be expected that SD may occur more frequently [7]. Several previous studies demonstrated a triphasic probability curve of SD versus MAP in which the probability of SD increased dramatically at the lower end of blood pressure, was flat at the middle range, and decreased further at the high end of blood pressure [7, 26]. This relationship follows the characteristics of the autoregulatory curve [16].

We previously assessed a small group of patients with aSAH who all had simultaneous monitoring of multiple different autoregulation indices to assess both agreement among those indices and relative predictive value for clinical outcomes and occurrence of SD [7]. In that study, we found that pressure reactivity (PRx) (derived from ICP), oxygen reactivity (ORx) (derived from PbtO_2_), and OSRx (derived from scalp near infrared spectroscopy [NIRS]) seemed to have the most consistent association with SD and possibly with clinical outcomes, though the study lacked power. In this current study, we expanded this cohort to a much larger data set and performed a more rigorous high-resolution assessment of each autoregulation index to determine whether these indices were associated with worse clinical outcomes and if SD occurrence was the cause or the result of impaired autoregulation.

## Methods

### Study Participants

Study participants enrolled in multiple studies related to multimodality monitoring at our institution over a period of 12 years were pooled for the current study (UNM IRB# 10-159, 17-297, 20-390, and 21-044), and data on multimodality monitoring were collected on these patients. All of these were observational studies without research interventions related to SD or multimodal monitoring parameters. Study participants with aSAH who had placement of multimodality monitoring were included. The clinical criteria for placement of multimodal monitoring were any patient with symptomatic hydrocephalus on admission or need for cerebrospinal fluid diversion during a surgical procedure. The Hummingbird (IRRAS USA, San Diego, CA) system [30] was used in this entire cohort. This system consists of a single twist drill bolt with an external ventricular drain (EVD) with an integrated parenchymal monitor for continuous ICP measurement built into the catheter. There are one or two additional side ports that allowed for the placement of additional monitors, typically a PbtO_2_ monitor (Licox, Integra LifeSciences, Princeton, NJ) and a CBF monitor (Bowman Perfusion Monitor, Hemedex, Waltham, MA). Most monitors are placed contralateral to the site of aneurysm rupture in case surgical treatment is needed. All patients also had bilateral frontal NIRS (INVOS, Medtronic, Minneapolis, MN) placed for clinical management. Some patients undergoing surgical treatment had additional placement of a 1 × 6 subdural strip electrode over the region deemed to be at the highest risk of ischemia (e.g., temporal for middle cerebral artery aneurysms or frontal for anterior communicating aneurysms). All these parameters, in addition to systemic parameters, such as blood pressure, are monitored at the bedside in the Moberg CNS monitor (Natus, Middleton, WI). Although there were some changes in clinical practice over the years, the standard multimodal monitoring approach was used with minor variations for most patients during this window.

### Scoring

In our previous study, we retrospectively calculated low-resolution versions of the various autoregulation indices using 1-min binned data exported from the Moberg CNS as a 30-min rolling average of Pearson’s correlation coefficients between MAP and each potential CBF surrogate [7]. With recent upgrades to the CNS Envision software (Natus, Middleton, WI), the originally proposed method for calculating PRx [31] can be applied to each additional parameter using the original waveform data. Briefly, each index is calculated as the 10-s rolling average of Pearson’s correlation coefficients between the waveform level arterial line MAP and the input function of interest over a 5-min moving window. Per standard intensive care unit practice, the arterial line transducer was leveled at the right atrium. We used ICP from the parenchymal monitor to calculate parenchymal PRx, ICP from the EVD to calculate EVD PRx, CBF from the Bowman probe to calculate cerebral blood flow reactivity (CBFRx), PbtO_2_ from the Licox to calculate ORx, and cerebral regional hemoglobin oxygen saturation (rSO_2_) from the INVOS to calculate (cerebral)oxygen saturation reactivity (OSRx). The right and left NIRS probes were averaged to a single measurement. We calculated each of these continuous indices using the CNS Envision software for all the retrospective stored study participant data. None of these data were available at the time of intensive care unit management, so no therapies were specifically directed toward autoregulation parameters.

SD recordings were acquired and scored per international consensus guidelines [20]. Briefly, a standard platinum 1 × 6 electrode was placed in the subdural space at the time of surgery. Electrocorticography (ECoG) was recorded using a direct current (DC) amplifier (Moberg CNS) directly into the Moberg CNS system, linking these data to the other multimodal monitoring parameters [32]. DC recordings (high-pass filtered at 0.005 Hz to stabilize drift) were overlayed on high-frequency bandpass filtered data (0.5–50 Hz). A SD cluster includes all SDs in the same patient occurring within 1 h of a consecutive pair.

### Data Processing

**Fig. 1:**
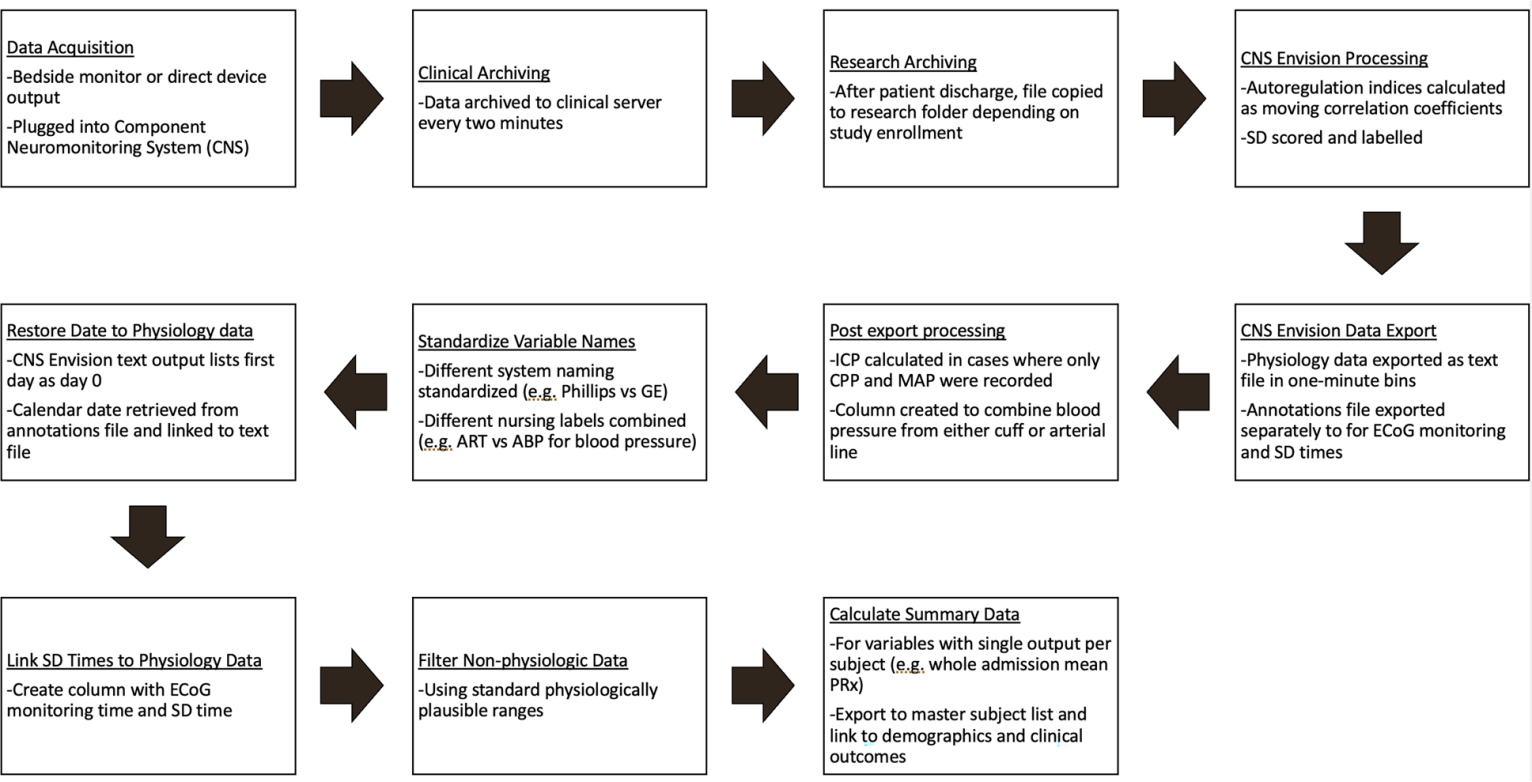
Workflow for data cleaning and analysis. ABP, ART, CPP cerebral perfusion pressure, ECoG electrocorticography, ICP intracranial pressure, MAP mean arterial pressure, SD spreading depolarization

The steps for data processing to a workable format required extensive development and are summarized in Fig. 1. As is noted, a large amount of processing was necessary to standardize measurements collected over many years of recordings. For example, several different bedside monitors were used, which required standardizing the naming. In these calculated parameters for autoregulation, the amount of variability in column titles or measurements across patients was increased further. Cases were examined on a case-by-case basis when needed to ensure accuracy of the standardized naming. All changes were made through coding rules to decrease any risk of errors and typographical errors in this process. Briefly, clinically recorded files were first copied from a clinical server to a research folder per institutional review board requirements. After calculation of autoregulation indices, data were then exported in text files in 1-min bins. Annotations including SD scoring and other events were exported as a separate file. Naming for variables was then standardized from various conventions that changed over the years. In cases in which the label was changed during the recording (e.g., ART changed to ABP), these were manually reviewed and then consolidated if it was determined to be the same data stream in that study participant. Results for complex cases were manually reviewed to ensure accuracy. In some study participants, there were multiple files that were relinked into one contiguous file. The recordings were then linked to the date and the times of SD. Data were then filtered to include only physiologically plausible ranges (e.g., to remove times when arterial lines were being accessed or “zeroed”).

### Clinical Data

Standard clinical variables were either recorded retrospectively with chart review or prospectively collected. Age, sex, admission diagnosis, admission Glasgow Coma Scale (GCS) score, admission World Federation of Neurosurgical Societies (WFNS) scale grade, and modified Fisher scale score were all collected. Angiographic vasospasm was recorded as the maximum severity recorded on the routine day 7 angiogram. Discharge and ~ 90-days modified Rankin scale (mRS) score were collected either from chart review or from prospective structured interview.

### Data Analysis

Summary values for each parameter of interest, in addition to clinical, demographic, and outcome data, were then compiled. The primary clinical outcome was discharge mRS score because this was the outcome parameter with the least missing data. Good outcome was defined as an mRS score of 0–3, whereas poor outcome was defined as an mRS score of 4–6. Two-tailed *t*-tests, as well as Mann–Whitney nonparametric tests, were used for continuous variables, and Fisher’s exact tests were used for binary variables. Study participants with missing data for a given variable were excluded from that analysis. Autoregulation indices were derived on the basis of Pearson’s correlation and hence are bounded between − 1 and 1. We applied Fisher’s transformation for correlation coefficients to the autoregulation indices before conducting *t*-tests for the difference between poor and good outcomes and pre-SD and post-SD trend analyses. Significance was considered at *p* < 0.05.

To assess the relationship between SD and blood pressure, probability curves were constructed using 20-min bins containing SD compared to bins without SD in reference to MAP. For this analysis, a new column that contained any blood pressure measurement (either from invasive or noninvasive sources) was created. Arterial pressure was prioritized, but if not available, noninvasive (cuff) blood pressures were carried forward to the next measurement. To determine the temporal relationship between SD and various autoregulation indices, we investigated the time trend of these indices pre-SD and post-SD. We first defined and identified SD clusters within a patient using the working definition described earlier. SDs that were more than 1 h before the first SD or 1 h after the last SD in a cluster were defined as belonging to different clusters. For each SD cluster, the pre-SD time series begins at 60 min before and ends at the first SD of the cluster, with a total of 60 bins, including the one for the first SD. The post-SD time series begins at the last SD of the cluster and ends 60 min later. These bins were nonoverlapping. We applied Fisher’s transformation of correlation coefficients and then conducted trend analyses on the transformed data using a linear mixed-effects model with random effects for individual patients as well as SD clusters. We conducted separate trend analyses for nonclusters of single SDs, clusters of multiple SDs, and the two pooled. We also explored other time boundaries, such as 2 h and 30 min, as well as varying length of the serial data. All processing and analyses were performed using Matlab, R, and Graphpad Prism (v10.1.1). The strengthening the reporting of observational studies in epidemiology (STROBE) reporting guideline tool was used for this observational study.

## Results

We identified 320 study participants meeting the inclusion criteria between 2010 and 2023. The mean age was 57 (standard deviation = 14). The median admission GCS score was 12 and WFNS scale grade was 3. The overall mean hospital length of stay was 24 days, consistent with these being a relatively poor-grade group of patients with aSAH. More patients (*n* = 198) underwent craniotomy or craniectomy, and the remainder (*n* = 122) underwent endovascular embolization. This trend has been changing with time as endovascular therapy has improved; however, surgical treatment was preferred especially early in the experience. Forty-seven of these study participants underwent SD monitoring.

Older age, lower admission GCS score, higher admission WFNS scale grade, and higher admission modified Fisher scale score were all associated with worse outcomes. Sex was not. Interestingly, angiographic vasospasm severity on the routine day 7–11 angiogram was also not associated with outcomes (see Table 1).

**Table 1:** Associations of patient characteristics, injury severities, and autoregulation indices with clinical outcome at discharge

	Good outcome at DC (mRS 0–3) *n* = 118	Poor outcome at DC (mRS 4–6) *n* = 202	*p* value	Test
Age, mean (StD), y	51.81 (12.26)	60.46 (13.46)	** < *0.0001***	*t*-test
Female sex, *n* (%)	70 (59.3)	139 (68.8)	0.0898	Fisher’s exact test
Admission GCS, median	15 (*n* = 29)	10 (*n* = 94)	** < *0.0001***	Mann–Whitney test
Admission WFNS, median	1 (*n* = 28)	4 (*n* = 87)	** < *0.0001***	Mann–Whitney test
Admission mFS, median	4 (*n* = 24)	4 (*n* = 58)	** < *0.0001***	Mann–Whitney test
Vasospasm (score = 0–3^a^), median	0 (*n* = 103)	0 (*n* = 157)	0.2991	Fisher’s exact test
PRx, EVD, mean (StD)	0.11 (0.10) (*n* = 68)	0.09 (0.13) (*n* = 122)	0.2546	*t*-test^b^
PRx, parenchymal, mean (StD)	0.17 (0.12) (*n* = 68)	0.18(0.14) (*n* = 132)	0.6869	*t*-test^b^
ORx, mean (StD)	0.03 (*n* = 95)	0.06 (*n* = 173)	***0.0005***	*t*-test^b^
OSRx (average R/L), mean (StD)	0.05 (*n* = 98)	0.12 (*n* = 167)	** < *0.0001***	*t*-test^b^
CBFRx, mean (StD)	0.25 (*n* = 66)	0.25 (*n* = 103)	0.9551	*t*-test^b^

CBFRx, cerebral blood flow reactivity, DC, discharge, EVD, external ventricular drain, GCS, Glasgow Coma Scale score, mFS, modified Fisher Scale, mRS, modified Rankin Scale, ORx, oxygen reactivity, OSRx, oxygen saturation reactivity, PRx, pressure reactivity, R/L, right/ left, StD, standard deviation, WFNS, World Federation of Neurosurgical Societies grade

^a^0 = no vasospasm, 3 = severe vasospasm

^b^For each autoregulation index, Fisher’s transformation of correlation coefficient was applied to the data before two-sample *t*-test

**Fig. 2:**
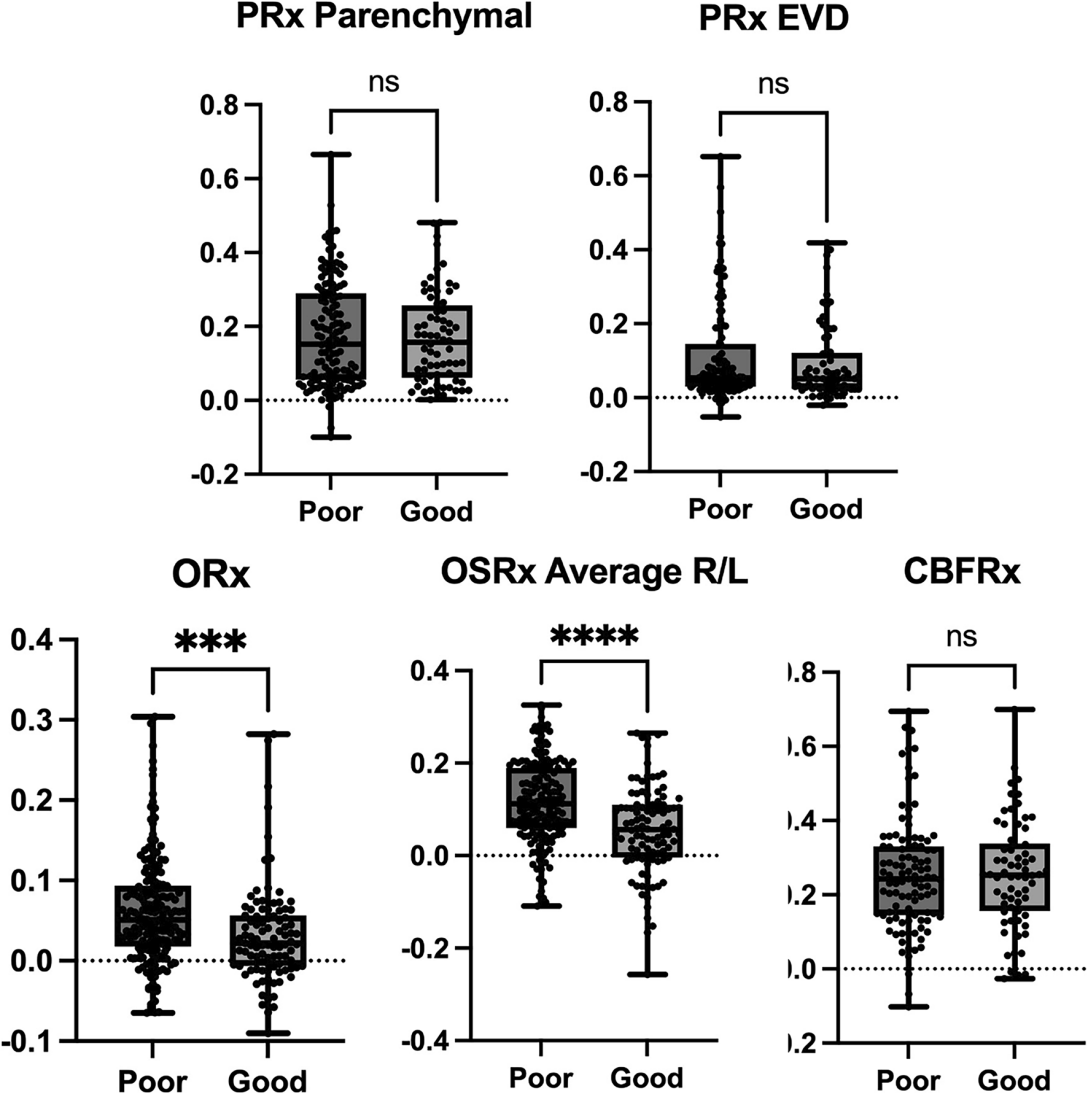
Box plots with raw data for each autoregulation index and clinical outcomes. CBFRx cerebral blood flow reactivity, EVD external ventricular drain, ns not significant, ORx oxygen reactivity, OSRx (cerebral)oxygen saturation reactivity, PRx pressure reactivity, R/L right/left for ABP

We hypothesized that impaired autoregulation (higher autoregulation index) would be associated with poor outcomes but found only ORx (*p* = 0.0005) and OSRx average (*p* < 0.0001) demonstrated a significant association with worse clinical outcomes (see Table 1 and Fig. 2).

**Fig. 3:**
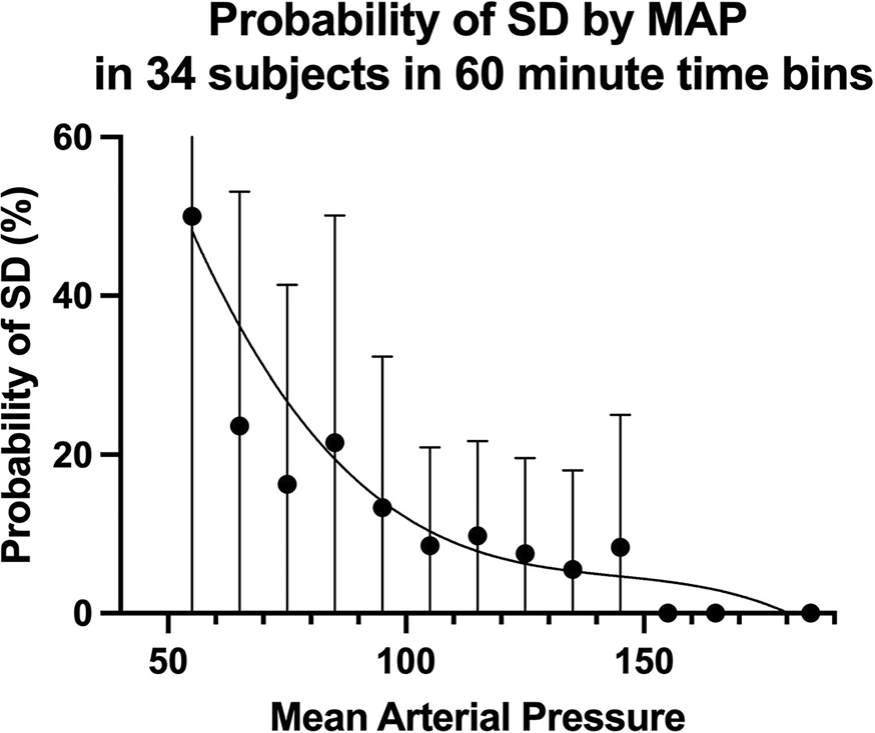
Relationship between probability of SD and MAP. This triphasic curve could plausibly be a reflection of a shifted autoregulation curve in this cohort. MAP mean arterial pressure, SD spreading depolarization

Plotting the probability of SD versus blood pressure, a familiar triphasic curve characteristic of CA was demonstrated [16] (Fig. 3). Thus, below an MAP of ~ 90 mm Hg, there was a nearly linear association of increased SD probability with decreased MAP. As noted in the plot, between 90 and 150 mm Hg, the probability of SD was relatively stable and low. Above an MAP of 150 mm Hg, no SDs were observed. These three transitions have previously proposed to represent a reflection of the autoregulatory curves and the upper and lower limits of autoregulation. In this cohort of poor-grade patients overall, a shift in the lower limit of autoregulation may be suggested.

**Fig. 4:**
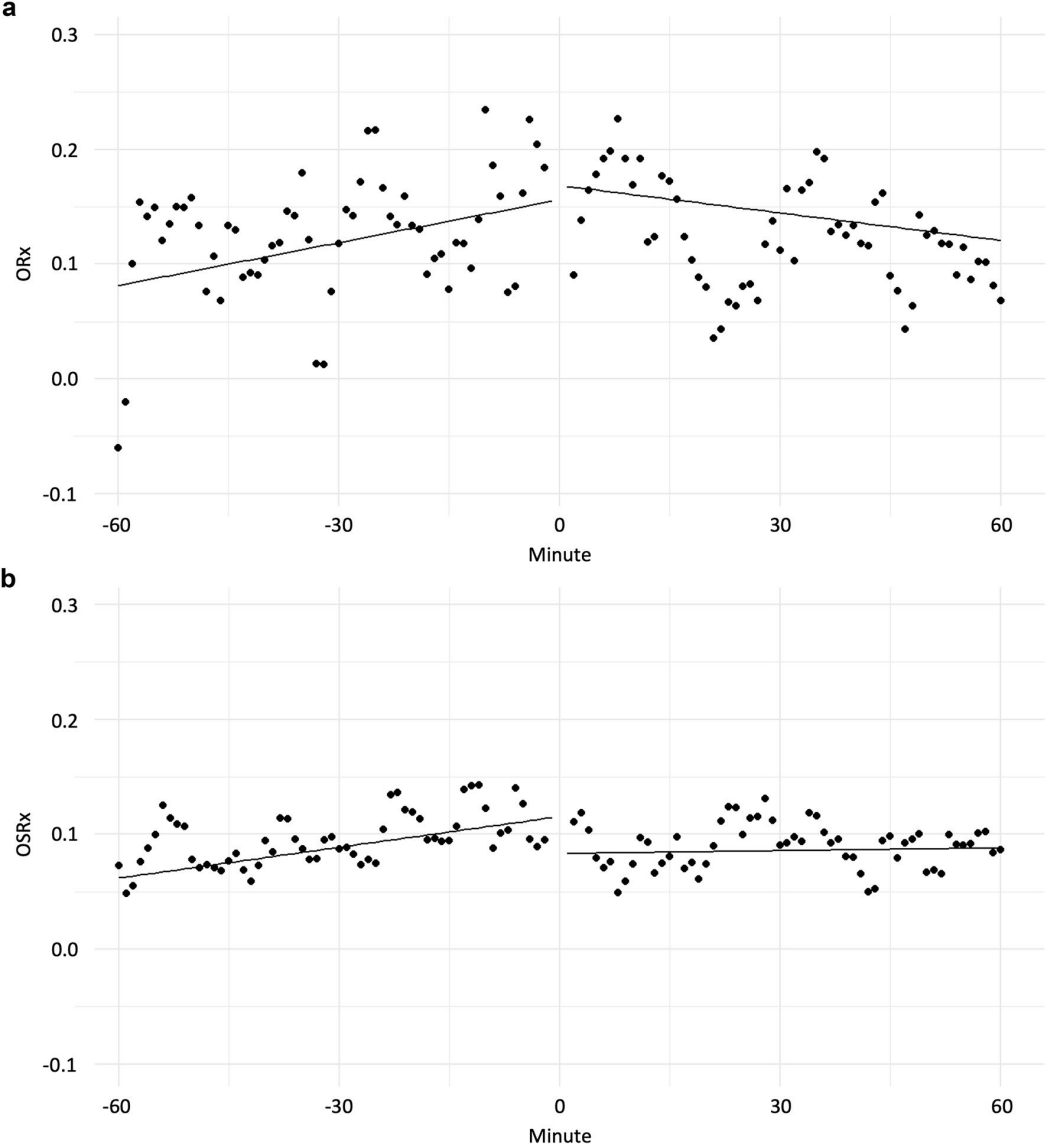
**a**, Trends in autoregulation index ORx in the 60 min pre-SD and 60 min post-SD. **b**, Trends in autoregulation index OSRx 60 min pre-SD and 60 min post-SD. Dots are 1-min bin average across all SD clusters. Lines are predicted mean values using a linear mixed-effects model based on serial data of individual SD clusters (see Table 2 for intercept and slope). Individual index values were first transformed using Fisher’s transformation for correlation coefficient. ORx oxygen reactivity, OSRx (cerebral)oxygen saturation reactivity, SD spreading depolarization

Regarding SD clusters, we found an increasing trend in ORx and OSRx average within 60 min just before SD clusters, as seen in the positive slopes derived from the fitted linear mixed-effects model (0.0013 [*p* = 0.001] for ORx and 0.0009 [*p* = 0.0001] for OSRx, respectively; Table 2) We also found a decreasing trend in ORx within 60 min just after SD clusters (− 0.0008, *p* = 0.0288) but no significant trend in OSRx post-SD clusters. Comparable and consistent results were seen when we analyzed single SD clusters and multiple SD clusters separately (see slopes in Table 2). Figure 4a, b displays this trend. This may suggest that worsening autoregulation measured by ORx and OSRx contributed to increased SD probability with potential improvement (e.g., in ORx) or stabilization (e.g., in OSRx) post-SD. Mean PRx from the parenchymal monitor was significantly higher prior to clustered SDs compared to isolated SDs but with a somewhat decreasing trend immediately before clustered SDs (slope =  − 0.0015, *p* < 0.0001) and increasing trend post isolated SDs (slope = 0.0008, *p* = 0.002). We also found a decreasing trend in EVD PRx prior to SD but an increasing trend post-SD with only isolated SDs. Significant pre-SD and post-SD trends were found in CBFRx only with clustered SDs not isolated SDs. It is interesting to note that the time trends in these autoregulation indices were robust with respect to time boundary, length of index serials, or removing time bins immediately proximate to SD clusters.

**Table 2:** Pre-SD and post-SD trend in autoregulation based on linear mixed-effects models fit to serial 1-min bin data up to 60 min before and after an SD cluster

Autoregulation index	Cluster	Pre-SD		Post-SD	
		Slope per min	*p* value	Slope per min	*p* value
PRx, EVD	Single SD	− 0.00049	***0.0349***	0.00068	***0.0014***
	Multiple SDs	− 0.00058	0.0556	0.00017	0.4866
	Pooled	− 0.00052	***0.0043***	0.00048	***0.0025***
PRx, parenchymal	Single SD	− 0.00040	0.1354	0.00083	***0.0021***
	Multiple SDs	− 0.00152	** < *0.0001***	− 0.00026	0.4324
	Pooled	− 0.00082	***0.0001***	0.00041	***0.0510***
ORx	Single SD	0.00124^a^	***0.0070***^a^	− 0.00009^a^	0.8376^a^
	Multiple SDs	0.00133^a^	***0.0463***^a^	− 0.00217^a^	***0.0007***^a^
	Pooled	0.00125^a^	***0.0010***^a^	− 0.00080^a^	***0.0288***^a^
OSRx (average R/L)	Single SD	0.00089^a^	***0.0017***^a^	− 0.00003	0.9265
	Multiple SDs	0.00088^a^	***0.0221***^a^	0.00029	0.4199
	Pooled	0.00089^a^	***0.0001***^a^	0.00008	0.7096
CBFRx	Single SD	0.00049	0.4094	0.00017	0.7835
	Multiple SDs	− 0.00195	***0.0249***	− 0.00202	***0.0062***
	Pooled	− 0.00036	0.4683	− 0.00058	0.2271

Bold/italic values are significant

CBFRx, cerebral blood flow reactivity, EVD, external ventricular drain, ORx, oxygen reactivity, OSRx, oxygen saturation reactivity, PRx, pressure reactivity, R/L, right/left for ABP, SD, spreading depolarization

^a^Consistent trends in direction of slope and significance

## Discussion

The importance of autoregulation in the management of aSAH has been previously explored as a strategy to develop individualized management strategies for patients at risk of ischemia related to DCI [33, 34]. Current American Heart Association guidelines for the management of aSAH [35] suggest that after ensuring appropriate euvolemia, permissive autoregulation strategies are reasonable; however, further validation of algorithms and real-time application are needed. In the absence of such individualized approaches, blood pressure augmentation in response to neurologic changes related to DCI may be considered [35]. The use of autoregulation-derived approaches potentially offers the opportunity to better refine such augmentation to the physiology of a given patient at a given stage after injury, though evidence for such strategies remains limited [36]. This is likely in part due to multiple different noninterchangeable methods to assess CA and a lack of clear understanding of the exact physiology that is being measured by these indices [37]. The current study therefore fills several important missing links in the literature. First, we compared several measurement approaches side by side in a relatively large cohort of patients and demonstrated consistent association with outcomes with ORx and OSRx, consistent with other reports in which the PRx was less strongly associated with outcomes in aSAH [6, 38]. Second, our data provides an important mechanistic link to outcomes based on the relationship of impaired autoregulation to SD.

These data serve as a validation of the hypotheses developed in a smaller cohort of patients who were only assessed if each study participant had all the autoregulation monitoring approaches (ICP, Partial pressure of brain tissue oxygen (PbtO_2_), CBF, and NIRS), including SD monitoring [7]. In that study, we found that oxygen reactivity (ORx), OSRx, and PRx were the most reliable indices in identifying the risk of worse outcomes in patients with aSAH. Only one of the two PRx measurements (parenchymal ICP) was associated with clinical outcomes. This PRx measurement was also inconsistently associated with SD occurrence. In the current study, PRx was not associated with outcomes, and there was a possible weak association with SD, though it was not consistent when considering single versus clustered SDs. On the other hand, both ORx and OSRx were associated with clinical outcomes and were found to increase prior to SD regardless of whether single or clustered events were considered. A strength therefore of the current analysis is that this larger study replicates and strengthens the results of the exploratory study.

Based on these data, we agree with current evolving management strategies that target MAPopt or CPPopt in patients with aSAH [33, 39] and also agree with the importance of tissue oxygenation as these studies have emphasized. The use of the ORx or OSRx as the input function for calculation of CPPopt or MAPopt may be a more effective strategy than using PRx and PbtO_2_ or rSO_2_ as separate measures to balance. Certainly, further practical studies are needed to determine the feasibility of incorporating such approaches at the bedside; however, our work demonstrating this potential application of either invasive or noninvasive approaches may facilitate use in more patients. The exact mechanism for this association is unclear, even if consistent with previously published data. One explanation could be that oxygen-based parameters are a closer approximation of CBF (the actual input function for autoregulation) as compared to ICP, especially in patients who are having ICP manipulations, such as drainage or craniectomy. If this is true, one might expect that CBFRx would be an even better predictor; however, we suspect that the relatively limited valid data time and frequent recalibrations of the Bowman CBF system may limit the assessment. Both the PbtO_2_ and NIRS measurements are relatively continuous measurements for multiple days with little data loss.

The second important link provided by our current analysis is in refining the relationship between autoregulation and SD. Specifically, SD triggered by decreasing CBF and impaired autoregulation may be one of the central mechanisms of secondary ischemia in aSAH. In a recent multicenter study from Europe, the peak total SD-induced depression duration of a recording day was found to predict ischemia and infarction with 60- and 180-min duration, and the authors concluded that SD was an independent mechanistic biomarker for DCI and delayed infarction in aSAH [13]. This is consistent with previous literature linking SD and spreading ischemia to worse outcomes in aSAH [13, 23]. In the current study, we once again identified a triphasic probability curve of SD, which has now been demonstrated in patients with traumatic brain injury [40] and ischemic stroke [26]. Overall, it appears that there is increased risk of SD (lower limit of autoregulation) as high as an MAP of 90–100 mm Hg in this population; however, we do not propose that this be used as in indiscriminate target. Instead, the use of the combination of autoregulation and SD monitoring could play an important role in better understanding the risk that a given patient may be at in terms of ischemia. For example, the occurrence of SD may be an indicator of metabolically compromised tissue (at risk of DCI), and progressively worsening duration of the ECoG depression could indicate the need to further optimize physiologic targets to avoid ischemia using MAPopt or other approaches [16].

Previous data on the relationship between SD and autoregulation was summarized in a recent comprehensive review [16]. In addition to the associations of ORx and OSRx that we previously reported [7], another group found associations between SD and impaired autoregulation as measured by the PRx [10], hypothesizing that SD may in fact be the source of impaired autoregulation. Interestingly, these findings were different depending on whether a parenchymal or ventricular source of ICP monitoring was used. In our data, PRx values from both the parenchymal and ventricular source demonstrated an inconsistent relationship with SD. Both tended to decrease prior to SD and generally tended to increase after SD, with significant positive slopes for both sources. These PRx trends indicate variability in relationship to SD but could be consistent with the previous associations of SD leading to worsening PRx [10]. Because PRx was not consistently associated with outcome, it seems that the association between ORx and OSRx with SD may be more clinically relevant.

The relationship between SD and autoregulation may also be more complex, as locally impaired autoregulation at the time of SD has also been observed [11, 41]. Therefore, SD may be triggered by globally impaired autoregulation and may further worsen autoregulation in the region of SD as one mechanism of ongoing tissue metabolic stress. This potentially cyclical relationship is therefore one explanation for these seemingly contradictory findings. In other words, it is possible that globally impaired autoregulation could be a risk factor for triggering SD due to metabolic supply demand mismatch. We think that our time series data demonstrating worsening autoregulation preceding SD supports this hypothesis. It is also highly probable that the SD itself causes local disturbance in autoregulation and that due to the location of our probes, it may not have been detected. Further analysis with probes placed adjacent to SD monitoring electrodes at the time of surgery may help clarify these issues.

Finally, given that there may be age-dependent changes in risk of SD [42], future analyses will focus on how age, sex, or other demographic factors may increase the risk of impaired autoregulation.

Although there are notable strengths to this study, including high-resolution recordings over a long period of time in a large relatively homogeneous cohort of patients with aSAH, there are clearly some limitations. Not all study participants had all parameters measured because of practice and technology changes over the course of monitoring. A relatively small number of only surgical patients had SD monitoring, so it is unknown if our observations related to SD apply to nonsurgical patients as well; however, it seems plausible that similar pathophysiology is at play. In addition, there is a risk that the continuous indices do not reflect the tissue most at risk for SD because the monitors used to generate these indices are typically placed contralateral to the surgical site and therefore the SD monitoring electrode site. Future studies assessing right versus left OSRx as well as alternate PbtO_2_ monitor placement will help understand the strength and cause and effect of these measurements as they relate to SD. Though the majority of patients had craniotomy only, 20 study participants underwent craniectomy. Previous data have suggested that PRx at least may be less reliable in such cases [31]. Further analysis will be needed to determine whether these trends remain in such patients. Finally, because this was primarily a cohort of more severely injured study participants requiring multimodality monitoring, the applicability to lower-grade patients is unknown.

## Conclusions

Impairment in CA in aSAH is most associated with worse clinical outcomes and occurrence of SD when using ORx and OSRx. Impaired autoregulation appears to precede SD occurrence rather than be a result of SD occurrence, though this could be a cyclical relationship. Targeting the MAPopt or CPPopt in patients with aSAH should use ORx and/or OSRx as the input function rather than ICP. The combined use of continuous autoregulation monitoring to determine the optimum blood pressure target with monitoring of SD as a mechanism of ongoing metabolic stress and risk of ischemia may be a rational strategy to improve outcomes in patients with aSAH.
